# Genome sequencing identifies *KMT2E*‐disrupting cryptic structural variant in a female with O'Donnell‐Luria‐Rodan syndrome

**DOI:** 10.1111/cge.14355

**Published:** 2023-05-08

**Authors:** Mona Hashim, Helen Stewart, Jing Yu, Benito Banos‐Pinero, Genomics England Research Consortium, Alistair T. Pagnamenta, Jenny C. Taylor

**Affiliations:** ^1^ Oxford NIHR Biomedical Research Centre, Wellcome Centre for Human Genetics University of Oxford Oxford UK; ^2^ Oxford Centre for Genomic Medicine Oxford University Hospitals NHS Foundation Trust Oxford UK; ^3^ Oxford Genetics Laboratories Oxford University Hospitals NHS Foundation Trust Oxford UK

**Keywords:** cryptic, genome‐sequencing, *KMT2E*, structural variant

## Abstract

We describe a patient from the 100,000 Genomes Project with a complex *de novo* structural variant within *KMT2E* leading to O'Donnell‐Luria‐Rodan syndrome. This case expands the mutational spectrum for this syndrome and highlights the importance of revisiting unsolved cases using better SV prioritisation tools and updated gene panels.

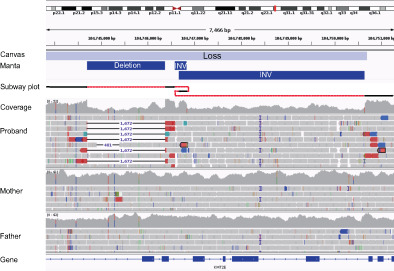

This first‐born child of healthy unrelated white British parents was born at 38 weeks weighing 3.67 kg by ventouse delivery following an uncomplicated pregnancy. Neonatal jaundice was successfully treated with phototherapy.

Mild unilateral talipes did not require treatment and congenital hip dislocation was treated with a harness from 5–10 months. Developmental milestones were delayed, sitting at 12 months, walking at 21 months with poor coordination, and with speech delay. Aged 11 she was ~3 years behind her peers with autistic traits. She attended mainstream school and college with educational support. She did not have seizures.

She had postnatal macrocephaly (occipitofrontal head circumference (OFC): 50th centile at birth, 98th centile at 9 months, 95th centile at 18 years). Her height was above 90th centile (91–98th centile aged 11; 98–99.6th centile aged 18). A large tongue was noted aged 11, broad forehead and square nasal tip at 18 years old, and the patient complained of constipation.

Array‐CGH analysis (Agilent ISCA‐60 K) identified a rare, paternally inherited 2.1 Mb 9q22.2q22.31 duplication. Of five genes involved, *SYK* and *AUH* are OMIM morbid genes (#619381, #250950) and neither fitted with clinical presentation. The large OFC at 11 years prompted Sanger sequencing and MLPA analysis of *PTEN* but no pathogenic variants were identified.

Given the uncertain significance of the 9q22 duplication, proband and parents had whole genome sequencing (WGS) through the 100 K Genomes Project, which seeks to embed WGS into NHS.^1^ Ethics approval was from Cambridge South REC (14/EE/1112). An in‐silico panel for Intellectual disability (https://panelapp.genomicsengland.co.uk/panels/285/, v1.158) was applied.

Analysis of WGS data confirmed the paternally inherited tandem‐repeat duplication (chr9:92010618‐94166278; GRCh37) and identified compound‐heterozygous variants in *SLC17A5*, linked to Salla disease (OMIM#604369). The maternally inherited NM_012434.5; c.406A>G; p.(Lys136Glu) is a well‐established pathogenic variant (VCV000021493.26). However, the paternally inherited c.85G>A; p.(Ala29Thr) is likely benign (VCV000781754.25), therefore, unlikely to be causative. This was confirmed by normal levels of urinary sialic acid in our patient.

Since the phenotype remains unexplained, we continued to investigate other variant types, including structural variants (SVs) which are under‐explored by the Genomics England pipeline. We applied SVRare^2^ which aggregates SVs in MySQL database and generates family‐specific and gene‐centric reports.

Our analysis identified several adjacent *KMT2E‐*disrupting SVs in the proband. Manta identified two inversions and a deletion, while Canvas called 7705 bp deletion. Closer inspection using IGV (v2.11.9) suggested a complex structure, with three deletions removing exons 18–24 (NM_182931.3) and two retained internal segments including part of exon19, one of which is inverted (Figure 1A). Although the breakpoints lie downstream of the SET domain, several other reported truncating variants lie further towards the 3′‐end of the gene.^3^

**FIGURE 1:**
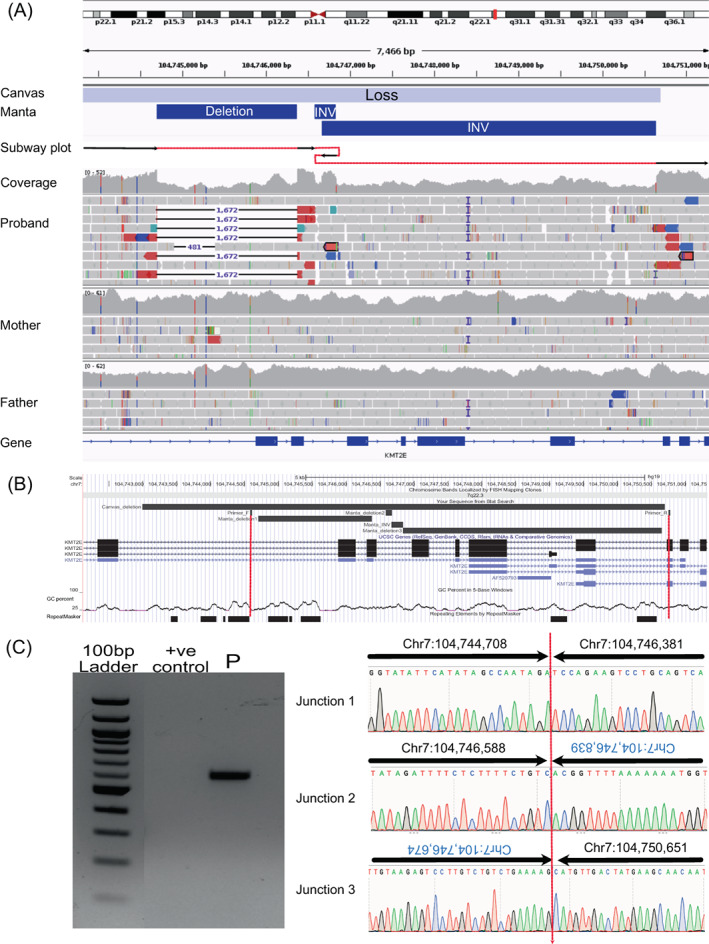
Discovery and validation of cryptic *KMT2E*‐disrupting variant. (A) Short‐read alignments showing complex *de novo* DEL‐DEL‐INV‐DEL called by Canvas/Manta. Neither tool captures the full complexity of the variant. (B) UCSC browser image showing primers (http://hgw1.soe.ucsc.edu/s/mhashim/KMT2E). (C) Unique band in proband but not in control. Sanger sequence of junctions confirming the identified SVs are a single event. Arrows indicate the direction of primers. Position numbers in blue indicate antisense sequences of the inversion. [Colour figure can be viewed at wileyonlinelibrary.com]

As part of an international collaboration in 2019, we initially described variants in *KMT2E* in 38 cases from 36 families with neurodevelopmental syndrome.^4^ This was followed by a second cohort^3^ and several reports, totalling >60 patients within the first 2 years of publication. The syndrome was subsequently named O'Donnell‐Luria‐Rodan syndrome (ODLURO; OMIM #618512). Most patients had mild intellectual disability, and 25% had autism. About 20% of patients with LoF variants had easy‐to‐control seizures. The clinical features of our proband were in keeping with ODLURO. To date, all LoF variants described are frameshift, splicing variants, nonsense, microdeletions of *KMT2E* or part of a larger complex SV.^5^ However, no cryptic SV within *KMT2E* has been described.

To validate this SV, we captured all junctions in a single PCR (Figure 1B). It verified the configuration of the DEL‐DEL‐INV‐DEL confirming it to be a single *de novo* event (Figure 1C).

This case is the first cryptic SV within *KMT2E* with a size smaller than expected to be detected by array‐CGH. The genomic context surrounding the variant presents a challenge for validation due to high GC content and multiple repeat regions, especially at the proximal end (Figure 1B). The overestimation of deletion length by Canvas and miscalling deletion as an inversion by Manta potentially confounded primer design. Visual inspection of paired‐read alignments was thus critical to successful validation.

This case highlights the importance of revisiting unsolved cases using better SV prioritisation tools and updated gene panels. This is evident as the original *KMT2E* study was published 3 years after the initial analysis of the proband's WGS data. Finally, there is an ongoing need for comprehensive assessment of SVs in clinical WGS programmes to ensure cryptic/complex ones are not overlooked.

## CONFLICT OF INTEREST STATEMENT

The authors declare no conflicts of interest.

### PEER REVIEW

The peer review history for this article is available at https://www.webofscience.com/api/gateway/wos/peer-review/10.1111/cge.14355.

## Data Availability

Data is in the National Genomic Research Library (doi: https://doi.org/10.6084/m9.figshare.4530893.v7).
